# Implementation of Quantitative and Systems Pharmacology in Large Pharma

**DOI:** 10.1038/psp.2014.40

**Published:** 2014-10-22

**Authors:** S A G Visser, D P de Alwis, T Kerbusch, J A Stone, S R B Allerheiligen

**Affiliations:** 1 Quantitative Pharmacology and Pharmacometrics, Merck Research Labs, Merck & Co, Rahway, New Jersey, USA; 2 Quantitive Pharmacology and Pharmacometrics, MSD, Oss, The Netherlands

## Abstract

Quantitative and systems pharmacology concepts and tools are the foundation of the model-informed drug development paradigm at Merck for integrating knowledge, enabling decisions, and enhancing submissions. Rigorous prioritization of modeling and simulation activities has enabled key drug development decisions and led to a high return on investment through significant cost avoidance. Critical factors for the successful implementation, examples on impact on decision making with associated return of investment, and drivers for continued success are discussed.

## Quantitative and Systems Pharmacology in Drug Discovery and Development

Systematic application of a model-based drug development paradigm has been identified as a valuable approach to reverse the continued decline of R&D productivity by shifting compound attrition from late clinical development to earlier stages, ensuring more robustly designed studies, delivering against go/no go criteria, and improving confidence in the compound.^1,2,3,4,5,6^ Model-based (or better stated as model-informed)^7^ drug development encompasses the utilization of a “quantitative toolkit” in clinical development that includes pharmacokinetic–pharmacodynamic models, disease models, comparator models, model-based meta-analysis approaches, trial design simulations, quantitative decision criteria, and performance metrics.^1,2,5^ More recently, the application area of model-based analyses has expanded into drug discovery and commercialization in by shifting the balance from traditionally used tools such as empirical (population) pharmacokinetics (PK) and pharmacodynamics (PD) models, toward quantitative and systems pharmacology and real-world evidence models.^3,5,8,9,10,11,12,13^

Quantitative and systems pharmacology has been defined by the National Institutes of Health as an approach to translational medicine that combines computational and experimental methods to elucidate, validate, and apply new pharmacological concepts to the development and use of small molecule and biologic drugs.^8^ A unique feature of quantitative and systems pharmacology is that it not only provides an integrated system-level approach to determining mechanism of action of new and existing drugs in preclinical and animal models and in patients, but also enables prediction of efficacy and safety of compounds with (novel) mechanisms of action at all stages of drug discovery and development. This emerging discipline has drawn an increasing awareness and focus in recent years. Two National Institutes of Health–sponsored workshops have been held; a white paper was released^8^; funding opportunities have been established^14^; and a scientific journal was launched.^15^

Advancements in the computational and experimental techniques have enabled largely systems pharmacology model development within academia, research institutes, and/or specialized technical companies. A focus on the development of system-level (mathematical) models through the integration of experimental data and knowledge has led to an improved understanding of human biology, pharmacology, and safety in multiple disease areas.^16,17,18^ For example, comprehensive cardiac models are developed that are tailored to the prediction of cardiac liability (see for overview).^19,20,21^ Within oncology, systems pharmacology approaches were used in quantifying anticancer drug synergy in resistant cells,^22^ and for predicting the effect of combination schedules on xenograft tumors.^23^ In the diabetes field, a recent review summarized the model contributions over the past five decades.^24^ This mathematical modeling, tightly linked to experiments, has had a great impact on the understanding of glucose homeostasis, diabetic condition, and its associated complications. Integration of diabetes modeling efforts also enabled new insights in the underlying mechanisms involved.^24,25^ Moreover, it highlighted the areas where more focused research is required. The application of systems toxicology modeling in drug safety assessment was illustrated by Hoeng.^26^

Within the drug industry, quantitative and systems pharmacology has a large emphasis on the application: the integration of knowledge in order to enable decisions and enhane submissions. A wide variety of drug development decisions are amenable to being informed by quantitative systems pharmacology. Examples include: go/no go decisions, dose and schedule selections, optimal trial designs and analysis, comparator differentiation, risk-benefit analysis, discovery target choices, hypothesis testing in *in silico* models, comparing drug candidate efficacy/safety profiles, predicting human doses, optimal combinations of compounds, and understanding the right patient population. In the submission and life cycle management phase, quantitative and systems pharmacology can support dose justification, bridge between ethnic groups, inform label sections, underpin commercialization strategy, and enable line-extensions.

**Figure 1:**
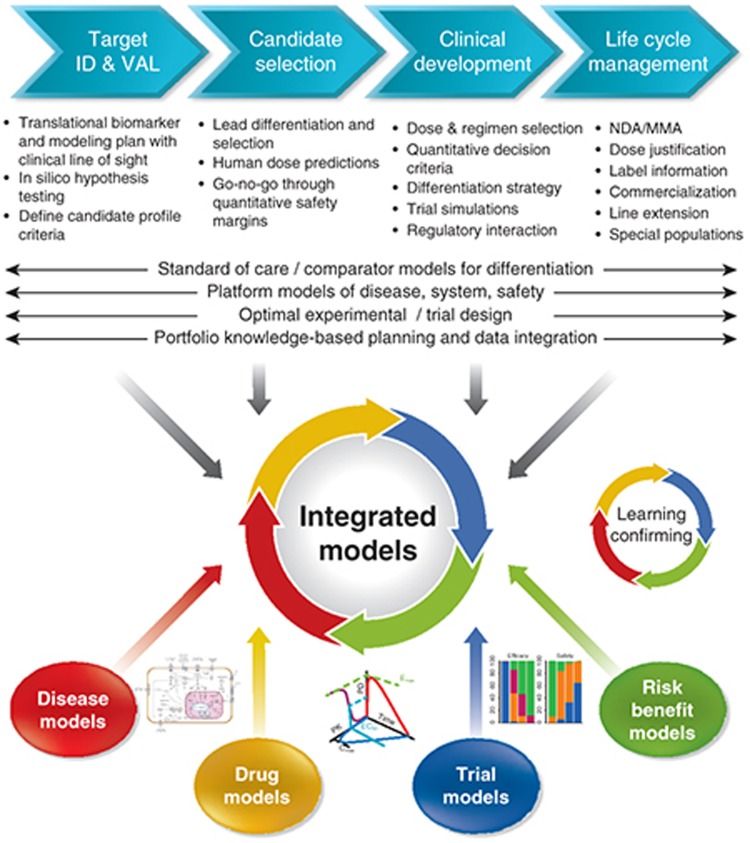
Within drug industry, quantitative and systems pharmacology focuses on the application: knowledge integration to enable decisions and enhance submissions. The aspiration and benefits of applying quantitative and systems pharmacology value are illustrated in the drug discovery and development continuum. Quantitative and systems pharmacology can be seen as the framework that focuses on the development of integrated models using the modeling toolbox that exists within quantitative sciences, supporting the model-informed drug discovery and development paradigm.

In light of its application, the degree of model complexity is determined by available information and the specific question that needs to be addressed, resulting in a continuum of models ranging from empirical to fully mechanistic models, with often a mixed “middle out” approach.^11^ In the pharmaceutical industry context, quantitative and systems pharmacology can, therefore, be considered as the framework that provides the full modeling toolbox that exists within quantitative sciences, supporting the model-informed drug discovery and development paradigm. This modeling toolbox includes models all the way from empirical (e.g., compartmental PK, dose–exposure–response models), (semi-) mechanistic (e.g., turn-over models, TMDD), tocomprehensive multidisciplinary (translational) systems models (e.g., ordinary differential equations, finite element method, logic-based, signaling network reconstruction, Bayesian model delineation) integrating biological, pharmacological, and clinical knowledge (**Figure 1**). In this respect, it should be emphasized that systems pharmacology models in an industry setting focus primarily on understanding of the biological pathway/mechanisms of interest and translatable pharmacological pathway interventions that are scalable to humans. Whereas understanding basic PKPD concepts (target site exposure, occupancy, and modulation)^4^ can enhance the confidence in the compound, systems pharmacology can increase the understanding of efficacy and adverse events in specific diseases and patient populations. In this way, quantitative and systems pharmacology enhances confidence in Proof of Concept, as was elegantly conceptualized by Vicini and Van der Graaf.^11^ For this reason, the primary focus of application of quantitative and systems pharmacology in drug discovery and development is to increase the probability of technical success in phase II. Some illustrations of impact examples using quantitative and systems pharmacology in industry are: decision on endometriosis dose management^27^; prediction of drug-induced modulation of human thyroid hormones based on dog toxicity data^28^; target selection in drug discovery^29^; understanding of complex PKPD behavior for bronchodilatory effect of zileuton, a 5-lipoxygenase inhibitor,^30^ reduction of the number of animals^31^; prediction of cardiac safety^32^; and *in silico* design of combination therapies and identification of predictive biomarkers in oncology.^33^

## Merck's Implementation Success Factors

**Figure 2:**
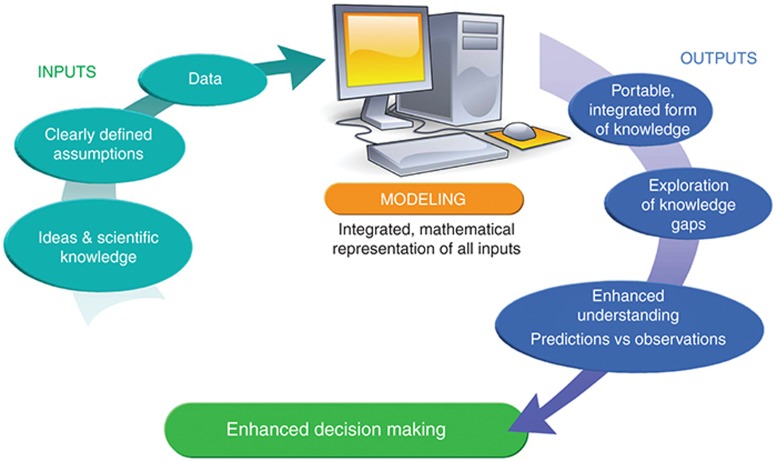
Quantitative and systems pharmacology model development generally follows a learning–confirming cycle. Key questions in a drug project are framed before start of model development, and the most appropriate modeling approach is identified. The model should reflect current physiological and statistical knowledge and is parameterized using data from various sources (ideas, literature, in-house or external studies, expert opinion, and assumptions). Transparency on the data, sources, and assumptions is critical. Previously unmeasured parameters can be identified, fit, or optimized on the basis of available data. The outputs are answers to the question at stake, enhanced understanding, and ability to explore untested scenarios through simulations. The model itself can be viewed as a representation of integrated form of knowledge.

Four years ago, Merck, among other pharmaceutical companies, recognized the need for the implementation of quantitative and systems pharmacology in drug development as a framework for the integration of quantitative knowledge in drug projects. The leading theme for implementation has been to provide timely quantitative answers to critical questions in the development projects in order to enable decision-making.^3^ Critical questions that are often posed in drug development teams are, for example: How much improvement is required in efficacy or safety to be best- or first-in-class?; What is the best dose?; Are there subsets of patients who respond differently?; Do we understand variability and uncertainty in critical biomarkers?; How does a biomarker relate to clinical outcome?; Can a human efficacious dose be predicted based on nonclinical results? To address these questions, experimentalists and quantitative scientists have been closely collaborating in developing and utilizing integrated models with the aim to enable key development decisions and to optimize patient outcomes. Many of the modeling efforts started out as focused projects for specifically defined questions and in incremental steps, evolved into more broadly applicable (systems) models leading to ongoing impact and value return (**Figure 2**).

**Figure 3:**
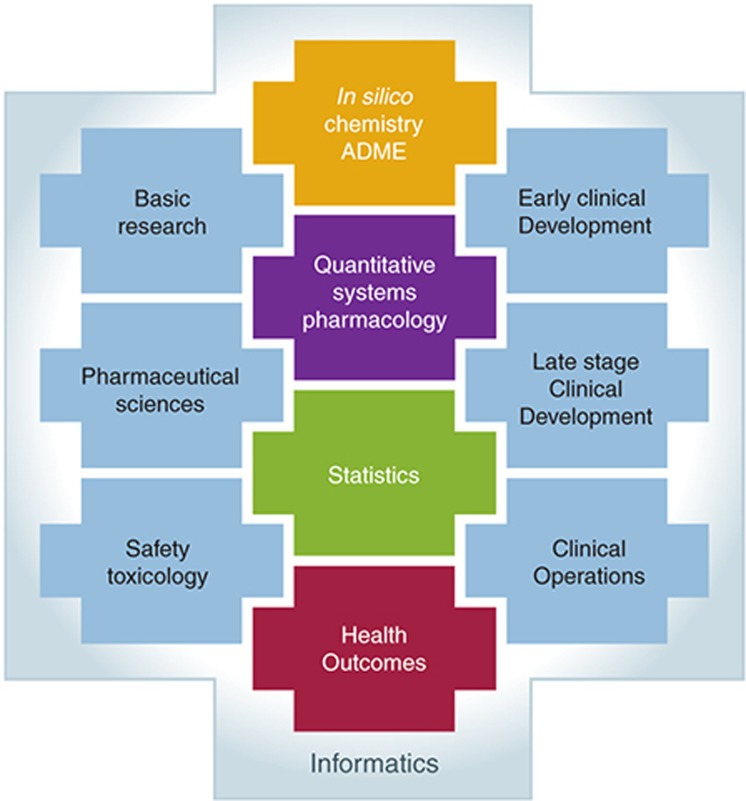
Quantitative and experimental functions require a collaborative network structure and empowered matrix leadership, irrespective of the organizational structure.

The time from project initiation to first impact on drug development can vary widely. It is dependent on the question of interest, project development phase, project timelines, and availability of the data. For modeling projects of clinical study data, the modeling and analysis plan typically is written directly after protocol finalization to be prepared for a quick turn-around of modeling results at interim analysis and/or after database lock. In these cases, the pace of the clinical study determines the total time for the modeling impact. However, for discovery and early clinical development project questions, such as translational understanding, systems and pathway analysis, and comparator modeling, time needs to be allocated for appropriate scoping of the background; gathering existing data and modeling approaches from literature, preclinical experiments, and previous studies; modeling of the data; interpreting and summarizing the results. It is not uncommon for a new modeling project that the data gathering and data curation into modeling ready datafiles can take more time than the modeling itself. However, within each therapeutic area, the quantitative strategy on the translational approach, systems and pathway analysis, and comparator modeling may be amenable for reuse in programs for related targets and back-up compound: and in such way have possibility for short lead times to impact. During the first 2 years, a modeling and simulation (M&S) capability group with a diversity of mathematical, conceptual, and matrix-leadership skills was established (backgrounds included: clinical pharmacology, pharmacy, pharmaceutical sciences, statistics, engineering, bioinformatics, mathematics, biology, and biochemistry). Most scientists were PhDs with varying level of experience (see also).^34^ This expert group recognized and responded to a flexible demand. Flexibility was obtained through partnerships with external vendors and through cross-functional network collaborations with other quantitative functions, such as biostatistics, informatics, applied mathematics, and clinical pharmacokinetics. Strong matrix leadership was a prerequisite for successful network collaborations and the optimal integration of experimental, clinical, and quantitative functional input (**Figure 3**).

Capability enhancement was achieved through process improvements, best practices implementations, and methodology development. One large component was the formation of a number of specialty modeling teams focusing on innovative methodology development and its application to the portfolio. A comparator modeling capability was built through the training of internal resources; training provided to project team members: development of uniform processes for data collection and warehousing; access to modeling tools and external vendors; and implementation of novel communication and visualization routines. Comparator modeling is now systematically used at Merck to differentiate compounds from standard-of-care treatment and (potential) competitors in order to define best-in-class criteria, determine clinical viability, and assist in marketing strategies. Another specialty team focused on improving clinical trial simulation methods and tools for trial design optimization from a cost avoidance perspective. In the formulation development area, *in silico* predictive tools and model-based IVIVC approaches supported formulation development and life-cycle management resulting in more successful late stage formulation trials and also regulatory success. Other specific focus areas for specialty teams included PK/QTc to assess QTc risk and inform trial design (thorough QT study, phase III QTc collection intensity), translational PKPD (informing probability of clinical success of preclinical candidates), pediatric, and physiological platform modeling. As for all these modeling efforts, the application to drug programs was imperative. In addition, infrastructure projects around computational capabilities were spearheaded in order to increase impact and efficiency. Finally, process improvements for reporting, quality control, reviewing and resourcing were put in place.

The key success factors in influencing decision making were the thorough scoping of opportunities and the rigorous prioritization of opportunities in each therapeutic area, enabling a maximized use of the M&S capability for activities with the highest value to the business. As seen everywhere, the opportunity space greatly exceeded the capacity and highlighted the need to focus on the highest value opportunities. Scoping and prioritization were facilitated by the formalizing M&S representation on development project teams and governance bodies. This single access point in the project team created a clear accountability for the quantitative systems pharmacology activities.

## Return of Investment and Enabled Decision Making

At Merck, the impact of quantitative and systems pharmacology in drug development has been demonstrated in many therapeutic areas in the form of dose selections (phase Ib, II, and III), study designs, go/no-go decisions, influences on clinical development plans and portfolio prioritizations at milestones (lead identification and optimization, preclinical candidate nomination, proof of concept, phase IIb and III and postmarketing). In addition, modeling supported decisions on dosing, formulation, pediatric, and thorough QT (studies; heart muscle repolarization test) (TQT) strategies in regulatory interactions and filings. This aligns with other industry reports on tangible impacts in terms of decision making, cost-savings, cost-avoidance, and cycle-time improvements.^5,12^

Merck has rigorously prioritized modeling support based on expected impact. Each year, ~50% of the opportunity space was prioritized. Approximately 10 projects were seen as informing key decisions. Impact was defined by senior management rather than by the modelers themselves. From the first year onwards, high impact was achieved if the model-informed decision resulted in a significant change in the program strategy which would have been unattainable without the modeling. The 10 key impacts annually typically were divided equally between enabling and no-go decisions. Among the M&S-enabled decisions were dose and schedule selection for phase I, II, or III, accelerated time to start of the next clinical trial and ability to file. In supporting regulatory interactions, M&S supported dose justification, bioequivalence claims, label recommendations, timing of TQT study and optimizing (costly) QTc assessments in phase III, and pediatric development plans and doses. No-go impacts were primarily due to quantitatively demonstrating the lack of a therapeutic window, or a too low probability of success for differentiation. As example: a drug development team asked if there existed a therapeutic window that would allow our molecule to be best-in-class. Integrated models were developed linking the relationship between the biomarker and the variability in exposure in order to understand the probability of preventing stroke while minimizing bleeding risk and QT prolongation potential. This was accomplished by leveraging an internal biomarker study, phase I and II trial results, and external data for hundreds of patients on comparator molecules. The modeling results indicated that there was no differentiation in safety vs. efficacy from existing comparators, and enabled the no-go decision. This no-go decision led to significant cost savings and demonstrated the high value of comparator modeling. The modeling-informed no-go decisions for these key impacts resulted each year in direct substantial cost avoidances for planned clinical trials as assessed by the finance department.

**Figure 4:**
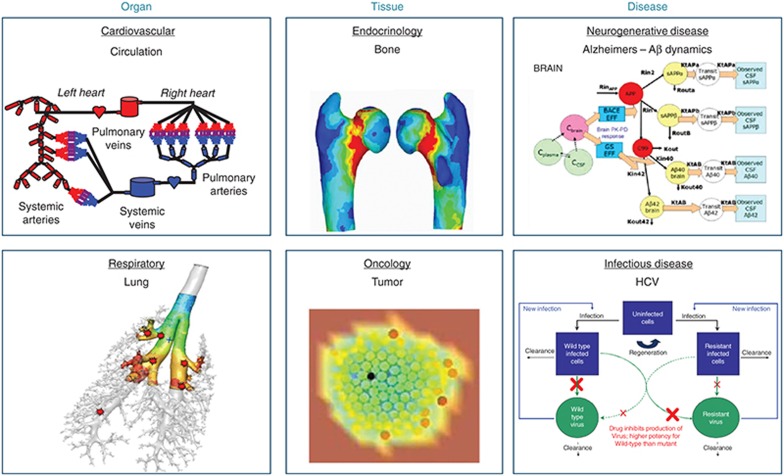
Illustration of Merck's variety of systems pharmacology models for virtual organs, tissue and diseases developed in house and with external partners. Courtesy of the virtual tumor graphic is Physiomics PLC.^23^

Two case studies are presented below to highlight the impact of quantitative and systems pharmacology on the development of new treatments for osteoporosis and psoriasis. The choice of case studies was made to illustrate the spectrum of enabling clinical decision making. The first case study is illustrating the application of finite element analysis to provide an *in silico* biomarker. The second case study is the application of a model-based meta analysis, as an illustration of the importance of our comparator analysis capability development. Beyond these examples, Merck strongly focused on the development of physiological platform models for organs (lung and circulation), tissues (bone- see case study below and tumors), and diseases (Alzheimers disease, hepatitis C virus, asthma, cancer, and diabetes), as illustrated in **Figure 4**. These *in silico* organs and systems disease models, being developed in house and in collaboration with external partners, are incrementally enhancing our internal drug discovery and development decisions with their ability to investigate what-if scenarios through simulations.

## Case Study 1: Finite Element Analysis of Bone Imaging Supporting Prediction of Clinical Efficacy of Odanacatib in Prevention of Postmenopausal Osteoporosis

Odanacatib, a selective CatK inhibitor, is currently being developed for the treatment of postmenopausal osteoporosis, a progressive degenerative bone disease leading to increased fracture risk. The associated morbidity and increased costs with the increase in life expectancy make osteoporosis an important world-wide health issue. Due to slow disease progression, large numbers of patients and long clinical trials are required to demonstrate the efficacy of new treatments that reduce fracture risk. The measurement of areal bone mineral density at the spine and hip is the current clinical standard for diagnosing osteoporosis, assessing the fracture risk, and estimating the treatment effects. However, the areal bone mineral density marker is a two-dimensional projection of the three-dimensional bone structure, and provides only a partial explanation of the bone fracture risk in osteoporosis patients. Full evaluation of bone micro- and macro-architecture in the assessment bone density can be done through high resolution-peripheral quantitative computed tomography, an *in vivo* imaging technique. Finite element analysis is used as an approach to mathematically recreate 3D structures based on the *in vivo* images and thereby aid in the evaluation of the bone mechanical response under various loading conditions and prediction of fracture risk. Therefore, finite element analysis could potentially serve as an improved noninvasive surrogate marker for bone strength and associated fracture risks.

**Figure 5:**
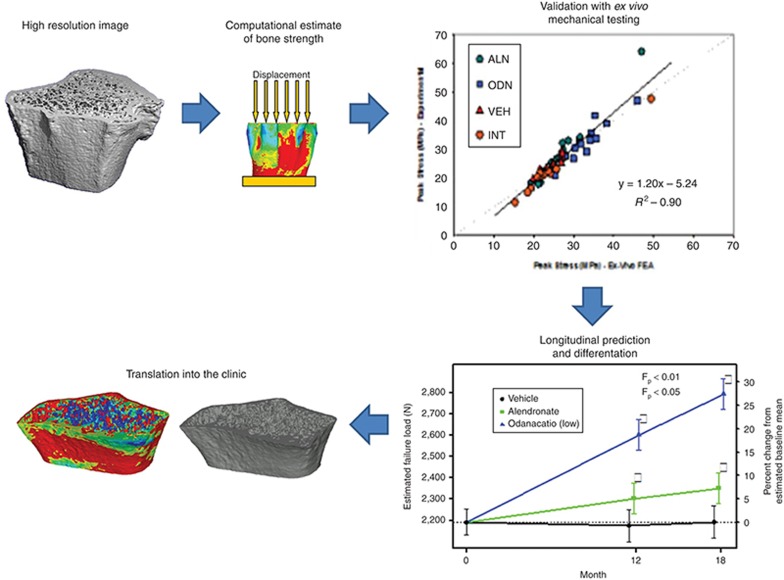
Schematic view of the development and qualification of finite element analysis as noninvasive marker for longitudinal prediction of bone strength and compound differentiation. From a high-resolution image of the ultradistal radius in a monkey, an engineering model of bone is created. Using finite element analysis, the deformations that the structure undergoes under a load are calculated. The force at which the bone goes into fracture is what is used to represent the strength of the bone. The finite element analysis was validated by comparing the predicted strength to actual failure load. Subsequently, high-resolution peripheral quantitative computed tomography and finite element analysis of bone strength at the distal radius in ovariectomized adult rhesus monkey demonstrated longitudinal efficacy of odanacatib and differentiation from alendronate. The finite element analysis method was incorporated in phase III trials to evaluate bone strength progression on the ultradistal radius site in humans.

Key questions in the odanacatib development program were: (i) Can finite element analysis be used as a noninvasive surrogate biomarker for bone strength and quality?; (ii) Can this marker support differentiation of odanacatib from standard-of-care treatment and thus provide insight in clinical viability; (iii) Can this biomarker, as exploratory endpoint in interim analysis, aid in prediction of clinical trial outcome to support go/no go decision? The finite-element-analysis-based bone strength methodology was developed and qualified to address the first two questions in the ovariectomized nonhuman primate, which is used as an animal model of postmenopausal bone loss. A schematic overview of the model qualification is provided in **Figure 5**. During *in vivo* studies, high-resolution bone images were taken to determine the impact of osteoporosis treatment on bone structure and strength. At the end of treatment, the bone sections were removed upon euthanasia, reimaged *ex vivo*, and experimentally tested for strength (peak load and stress) in axial compression using a hydraulic device. Based on the images, finite element analysis provided *in silico* predictions of these bone strength parameters. The qualification of the finite-element-analysis was confirmed by a good correlation between finite element analysis predictions and *ex vivo* measurements of bone strength with the understanding of its variability (For detailed description see refs. 35,36). Moreover, high-resolution imaging outperformed by 28% the classical X-ray-based bone mineral density as a fracture predictor.^35^ In a subsequent study, the predictive performance of finite element analysis was investigated and qualified through predictions of longitudinal bone changes and treatment efficacy based on images earlier in the study. Moreover, in a head-to-head comparison, the qualified finite element analysis method also quantified the superior efficacy of odanacatib over alendronate in ovariectomized nonhuman primates.^36^

Successful prospective predictions in nonhuman primates, coupled with experimental cadaver data from the literature, provided confidence that assessment of bone strength through finite element analysis in clinical studies could be used to support the third question. In a 2-year randomized, double-blind placebo-controlled phase III trial, finite element analysis was included as an exploratory endpoint for estimation of bone strength and longitudinal prediction of efficacy to support internal decision making.^37,38^

In summary, finite element analysis has been used as a noninvasive surrogate biomarker for bone strength and quality, and thereby has provided unique clinical insight into the biomechanical effects of new osteoporosis therapy, and has enabled comparator differentiation and clinical longitudinal predictions of bone strength, thereby enabling decision making in the odanacatib program.

## Case Study 2: Phase IIB and Phase III Dose Selection of Tildrakizumab for Treatment of Psoriasis Leveraging Comparator Data

Tildrakizumab is a monoclonal antibody designed for the treatment of psoriasis. Psoriasis is a common, chronic immune-mediated skin disease that varies in severity from minor localized patches to complete body coverage. Psoriasis Area and Severity Index (PASI) is the most widely used tool for the measurement of severity of psoriasis. PASI combines the assessment of the severity of lesions and the area affected into a single score in the range 0 (no disease) to 72 (maximal disease).

Key questions in the development program were: (i) What is the comparative efficacy of tildrakizumab against it competitors?; (ii) What doses need to be studied in phase IIb to establish the full dose response curve?; (iii) What is the optimal dose and schedule to be used for phase III confirmatory trials?

**Figure 6:**
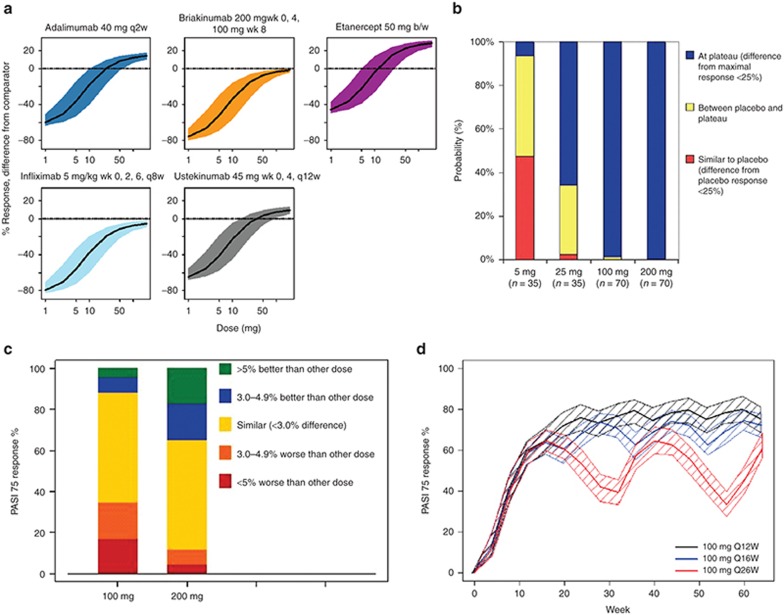
Dose selection of Tildrakizumab for treatment of psoriasis levering comparator data analysis. (**a**) Comparative landscaping: efficacy (y-axis) for various subcutaneous (SC) doses of tildrakizumab (based on Ph1b data) as compared to recommended dosing regimens for adalimumab, etanercept and infliximab, ustekinumab and briakinumab with 80% confidence intervals. (**b**) Results from clinical trial simulations for the selected dose range for phase IIb development of tildrakizumab. (**c**) Results from clinical trial simulations for PASI-75 in cohorts of *n* = 300 based on exposure–response models. (**d**) Clinical trial simulations of PASI-75 response rate during treatment with 100 mg SC tildrakizumab administered in 12-, 16-, or 26-week dosing intervals.

The first two questions were addressed in a comparative dose–response model analysis across five comparators (adalimumab, etanercept, infliximab, ustekinumab, and briakinumab, with published mean study-arm level data from >10,000 patients) and in-house phase Ib data of tildrakizumab.^39^ Tidrakizumab was compared against comparators assuming that all parameters, except for drug potencies, were similar across all comparators (**Figure 6a**). In clinical trial simulations, the doses were explored that would characterize the full-dose response curve. The doses 5, 25, 100, and 200 mg administered at weeks 0 and 4, followed by every 12 weeks were predicted to provide evidence of the plateau of the dose–response curve, to allow for of a dose–response relationship, and to enable determination of the lowest dose resulting in meaningful efficacy (**Figure 6b**).

The phase IIB study results were in accordance with the predictions made and subsequently used to develop an exposure–response model to analyze PASI-75 and PASI-90 response rates.^40^ Clinical trial simulations were conducted to investigate the potential outcome of phase III clinical trials. Key assumptions were made that (i) phase IIb study subjects were representative for the phase III population; (ii) the average serum concentration (*C*_avg_) up to week 16 was a reasonable measure of exposure to predict week 16 PASI-75 and PASI-90; and (iii) that the developed exposure–response model was appropriate to describe tildrakizumab responses. In total, 10,000 trials were simulated with cohorts of 300 subjects. Results demonstrated that the 100 and 200 mg doses are likely near or at the plateau of the exposure–response relationship (**Figure 6c**) and were both selected for phase III development to evaluate if the 200 mg dose may have a clinically meaningful higher PASI-90 response rate. To address the optimal scheduling question, a semimechanistic PK-PD model was developed to describe the PASI fraction of baseline over time.^41^ Clinical trial simulations were conducted to explore various maintenance doses and dosing frequencies taking parameter uncertainty and between-patient variability into account. **Figure 6d** shows that the 12-week dosing interval results in a sustained response at the 100 mg dose level. A less frequent dosing regimen would not lead to a sustained PASI-75 response. This underpinned the decision using a 12-week dosing frequency for 100 and 200 mg with the expectation to result in optimal efficacy for the treatment of psoriasis. In summary, model-based analysis, comparator analysis, trial simulations have been critical components in design and analysis considerations of tildrakizumab phase II and III clinical trials.

## Drivers for Continued and Systematic Success of Quantitative and Systems Pharmacology

Despite significant impact examples as provided above and found in the literature, continued efforts are required to reach the benefit potential of quantitative and systems pharmacology in drug discovery and clinical development. These specific focus areas include: effective communication of modeling results and impact; adequate qualification of systems pharmacology models; timely funding and efficient execution for the work to be influential; integrative knowledge enabling infrastructure; strategic partnering with external specialists: systematic implementation in drug discovery; and increased utilization in regulatory interactions.

Effective communication remains a challenge for modelers. On one hand, scientists need to be able to lay-out visionary messages on tangible benefits for approval on resourcing from senior management. On the other hand, they need to be able to develop, communicate, and review a sound technical approach. Moreover, successful model development requires substantial experimental data collection and strategic contributions from a variety of functional experts (e.g., pharmacology, biology, clinical research, biostatistics, mathematics). It can be challenging for the modeler to efficiently solicit biological and pharmacological input (scientific and experimental) without burdening the pharmacologist with mathematical and modeling details. In general, detailed technical and mathematical descriptions should be used only for documentation purposes and a peer-to-peer learning environment, and not for cross-functional or management discussions. However, model skeptics often require a high level of technical detail, which when provided often still is insufficient for reducing skepticism. Therefore, technically-skilled scientists possessing excellent communication skills are needed. It also may be helpful to pair technical experts with good communicators to lay out the application and value.

The qualification of models is essential in the use and communication of systems pharmacology models. The level of model qualification is dependent on the question being addressed. If a multimillion dollar clinical study is at stake, it requires significant confidence in the models' robustness. Robustness is not only assessed through the standard evaluation of parameter estimate precisions, diagnostic plots, individual fits, and predictive checks, but also through the evaluation of the models' consistency with the physiology and evaluation of parameter sensitivity to key assumptions. In addition, model robustness is tied closely to experimental data and can be enhanced by generating time-course data, integrating multiple data sources, evaluating prospective predictions of nonobvious responses, and validating through external data. In the recently proposed framework for model qualification of physiological models,^42^ the first step frames the project questions and the project team agrees on the use of the model inferences for the decision making. In a second step, the critical assumptions are summarized and highlighted. Moreover, data sources (clinical, preclinical, literature) and a method for assessing inter- and between-subject variability are clarified upfront. Following the modeling, uncertainty in predictions (e.g., sensitivity analysis for critical assumptions, data, and parameters) are assessed. To ensure biologically plausible results, communication of the approach (assumptions, limitations, results, and interpretation) with the relevant functions (biologists, clinical pharmacology and disease experts) is critical. This approach is not different from an earlier proposed general model qualification method to ensure that physiological models are fit for purpose.^43^ Beyond statistical evaluation, a physiology model can be used with confidence in drug discovery and development if it has addressed the following criteria: relevance to research context, dealing with uncertainty, dealing with variability, comparison to data.^43^

A team's failure to achieve the intended impact can be a result of getting stuck in the model building phase. Good planning dictates that model building be completed well in advance of a decision date, in order to allow for simulation, interpretation, review, and discussion with in the project team. Another failure of demonstrating timely impact can be that development of complex disease/systems pharmacology models requires substantial time and investment. Therefore, efforts should be made to rapidly develop critical components to answer specific project questions, thereby providing incremental value for the business prior to full integration into a comprehensive platform model. Again, visionary leadership and good communication is helpful to lay-out future benefits for prioritization and justification of investments. Moreover, continuous managing of key stakeholder expectations increases the likelihood of model-informed decision making.

Integrative understanding of preclinical and clinical data is imperative to enable informed decisions. Both the volume and variety of data generated during drug development have increased tremendously. Efficient use of modeling requires access to diverse and high quality data sets and a flexible data management infrastructure. Achieving this task in large pharmaceutical companies is challenging because the data sources and types differ among therapeutic areas, functional areas, and development stages. Without appropriate data capture, storage, and retrieval routines, data transformation, integration, and visualization will not be possible. One prerequisite is the availability of agreed data standards to allow integration of experimental data from various sources and a flexible data capture tool.^12^ In addition, visualization tools based on the knowledge plot concept^44^ would allow integration of *in vitro*, *in vivo*, clinical, efficacy, and safety data in order to enable scientific and informed decision-making in various stages of drug development. Such integration and visualization tool could be used as a forward- and back-translational tool, that can result in an improved understanding of the competitive edge for a particular project or disease area portfolio, and benchmarking multiple attributes evaluating compounds during due diligence.

Development of specialized physiological platforms models can occur via strategic partnering with vendors or institutions that provide advanced data-analysis tools, in depth (mathematical) knowledge of the disease, and/or specific technical expertise. The mutual benefits of such partnering include access to highly specialized models for pharmaceutical industry, and access to experimental data for specialized partners. These model-building collaborations often lead to new business opportunities (in the form of models, data sets, technology, software, etc.) for these partners that they could sell to others. Therefore, creation of collaboration agreements that ensure optimal exploitation of advanced models beyond the development phase is imperative. Also, it can be anticipated that the investment of large pharmaceutical companies in specialized model development through partnering with vendors, may eventually lead to future “off the shelf” purchase of platform model solutions. In such cases, due diligence would be required on the scientific foundation and on the ability to address project questions. Multiple modeling approaches to predict QT liability in the cardiovascular area illustrate the need for clarification on the qualification level for the intended application.^32,45,46,47^ To this end, it may be advantageous to precompetitively share data and knowledge for comparison and crossvalidation. In recent discussions with regulators, such appeal was made for the precompetitive sharing of models and data in collaborative efforts between pharmaceutical industry, academic investigators, research institutes (such as National Institutes of Health), and regulators.^18,48,49^ An example of such successful collaboration is the CAMD consortium focusing on understanding the placebo response in Alzheimer's disease clinical trials.^50^ A number of challenges in collaborative model building include definition of data standards, agreement on approval processes, and life-cycle management of the model and knowledge; however, the opportunity lies in the advancements in predicting drug safety and efficacy.

The benefit–risk relationship of a drug is largely determined once the target and compound are selected.^11^ Therefore, a systematic implementation of model based drug discovery has been advocated to increase the phase II proof of concept success rate.^4,9,12^ Successful utilization of quantitative and systems pharmacology (concepts, models, resources) in drug discovery requires a change from discrete functional contributions to a crossfunctional integration of knowledge in discovery project teams. Early alignment on a translational quantitative biomarker plan should provide the clinical line of sight for both biomarker development and modeling activities. Adaptive *in vivo* experimental design in the screening cascade can enable learning about the system properties of relevant markers, comparing to comparator compounds, and translation combined with early experimental medicine studies.^12,31^ Moreover, enabling technologies, such as real-time visualization and quick simulations prior to all *in vivo* experiments, could impact assumptions, trigger learning, and optimize use of animal resources, and enable rational integrative decision making on target and compound progression.

A last challenge is the use of model-informed approaches in regulatory interactions. Readiness and an increase in willingness from Regulators to accept model-informed approaches in the interactions with the sponsors have been noted at recent M&S workshops (in 2013 with the US Food and Drug Administration and in 2011 with the European Medicine Agency). However, given the potential complexity of systems pharmacology models and the level of inferences made, specific documentation requirements for this area should be clarified from a regulatory perspective. In recent discussions between European Federation of Pharmaceutical Industries and Associations and European Medicine Agency, a documentation framework was proposed, in which the level of scrutiny in modeling documentation is related to the importance of the decision at hand.^48^ In addition, regulatory acceptance of systems pharmacology model qualification is needed to ensure that integrated knowledge can assist in model-informed trial design and in extrapolations beyond the observed data range.^7^ Besides that, more importantly, the willingness (courage) is needed within the industry to make quantitative systems pharmacology model inferences part of the submission package, clinical trial applications, and scientific advice interactions. Early and continuous engagement with regulators from IND stage onwards is paramount for a mutual understanding and comfort in underlying assumptions for both the Sponsor and the Regulator allowing rapid regulatory decisions.

## Concluding Remarks and Future Perspectives

Merck's implementation of quantitative and system pharmacology in drug development focused on enabling key decisions through rigorous prioritization of opportunities, timely execution, influential communication, adequate model qualification, and securing team/management buy-in and funding. A high return on investment through significant cost avoidance was demonstrated. Therefore, we advocate a mechanistic and holistic approach, prioritized to impact key decisions in both drug discovery and development programs, using the diversity of tools that a quantitative and systems pharmacology approach offers. Integrated, comprehensive, multidisciplinary models should be developed through close collaboration between experimental and quantitative disciplines, with clear transparency of the critical assumptions and the impact of variability and uncertainty. One of the drivers for quantitative and systems pharmacology success is the access to the right skill combination. Scientists with highly developed communicative skills and an aptitude for project management should pair with mathematical and information-science experts for effective model development and engage regularly with biology and pharmacology experts. Furthermore, teams should be enabled in their dedication in time and resources to systems pharmacology model development beyond single compound development projects. A long-term value can be argued for industry, academia, and technology companies to collaborate in the design of experimental protocols that advance large-scale data collection (biochemical parameters and physiological outputs), and in the development of mathematical tools. The appropriate infrastructure for knowledge management (data capture, storage, processing and retrieval) is prerequisite to further enhance development of quantitative and systems pharmacology models as carriers for integrated knowledge across discovery/development continuum.

In conclusion, a systematic implementation can be claimed when a model-informed drug discovery and development paradigm becomes fully institutionalized to integrate knowledge, enable decision making, and enhance submissions. In such way, it builds for a future *in silico* drug-development, in which we can reduce *in vivo* and clinical drug testing and increase the number of effective treatments for patients. Moreover, these *in silico* models can be ultimately be used at the “bedside” to optimally inform patient and individual dose selection.

## Conflict of Interest

All authors are employees of Merck.
